# Conventional and complementary alternative medicine therapies for renal anemia: a literature review

**DOI:** 10.3389/fendo.2024.1342873

**Published:** 2025-01-22

**Authors:** Ching-Ming Lu, Yuan-Hsuan Hsu, I-Hsin Lin, Ko-Lin Kuo, Jian-Fu Liao, Hui-Fen Huang, Ping-Hsun Lu

**Affiliations:** ^1^ School of Post-Baccalaureate Chinese Medicine, Tzu Chi University, Hualien, Taiwan; ^2^ Department of Chinese Medicine, Taipei Tzu Chi Hospital, Buddhist Tzu Chi Medical Foundation, New Taipei, Taiwan; ^3^ Division of Nephrology, Taipei Tzu Chi Hospital, Buddhist Tzu Chi Medical Foundation, New Taipei, Taiwan; ^4^ Division of Nephrology, Tai An Hospital, Taipei, Taiwan

**Keywords:** chronic kidney disease, diet control, dietary supplement, complementary and alternative medicine, renal anemia, conventional medical therapy

## Abstract

Renal anemia stems mainly from chronic inflammation with elevated hepcidin levels, iron deficiency, and reduced red blood cell lifespan. Inadequate erythropoietin (EPO) production, worsened kidney function, leads to symptoms such as low energy, fatigue, and impaired physical function, significantly affecting patients’ quality of life. We conducted a comprehensive search across electronic databases including PubMed, Embase, Cochrane Library, Chinese National Knowledge Infrastructure, Airiti library, and Wanfang, to compile recent clinical trials and pilot studies on conventional and complementary alternative medicine approaches for renal anemia. This discussion focuses on the hypoxia-inducible factor prolyl hydroxylase domain (HIF-PHD) axis theory, from lab research to clinical applications. It explores non-extracorporeal treatments for renal anemia, including pharmaceutical interventions, dietary strategies, and complementary and alternative medicine (CAM). The article details the effects of Roxadustat, Ferumoxytol, and Epodion.

Clinical studies show that modulating the gut microbiome can reduce inflammation and improve renal anemia. Clinical trials suggest that CAM therapy can improve renal anemia through mechanisms such as enhanced iron metabolism, anti-inflammatory effects, reduced hepcidin levels, and increased EPO and HIF expressions. By synthesizing this information, the review aims to furnish valuable insights and treatment recommendations aimed at ameliorating renal anemia in individuals grappling with chronic kidney disease.

## 1 Introduction

Presently, chronic kidney disease (CKD) is delineated by markers of kidney impairment, involving imaging or proteinuria (typically assessed through the albumin-to-creatinine ratio: ACR) and diminished renal function (falling below the glomerular filtration rate: GFR thresholds, estimated from the serum creatinine concentration) (1–3). CKD constitutes a pervasive global health challenge, and its frequency has been on the rise (4, 5). As kidney function progressively declines, the prevalence and severity of anemia escalate. Sir Richard Bright initially recognized anemia as a common complication of CKD in 1836, noting a pallor in the facial complexion of patients with kidney ailments (6). Historically, anemia in CKD has been attributed to numerous symptoms accompanying the diminishing renal function, including fatigue, reduced strength, heightened dyspnea during exertion. Studies conducted earlier established a robust correlation between higher hemoglobin levels and various parameters reflecting better physical functioning and quality of life (QoL) in patients at stages 3, 4, and 5 of CKD (7–9). The incidence of anemia in individuals with CKD rises concomitantly with the decline in GFR. For instance, the prevalence of anemia climbs from 1% among patients at stage 3 CKD to 67% at stage 5 (10, 11). Furthermore, the presence of anemia in CKD is linked to an elevated risk of cardiovascular disease, left ventricular hypertrophy, increased hospitalizations, cognitive impairment, and heightened mortality (12, 13).

**Figure 1:**
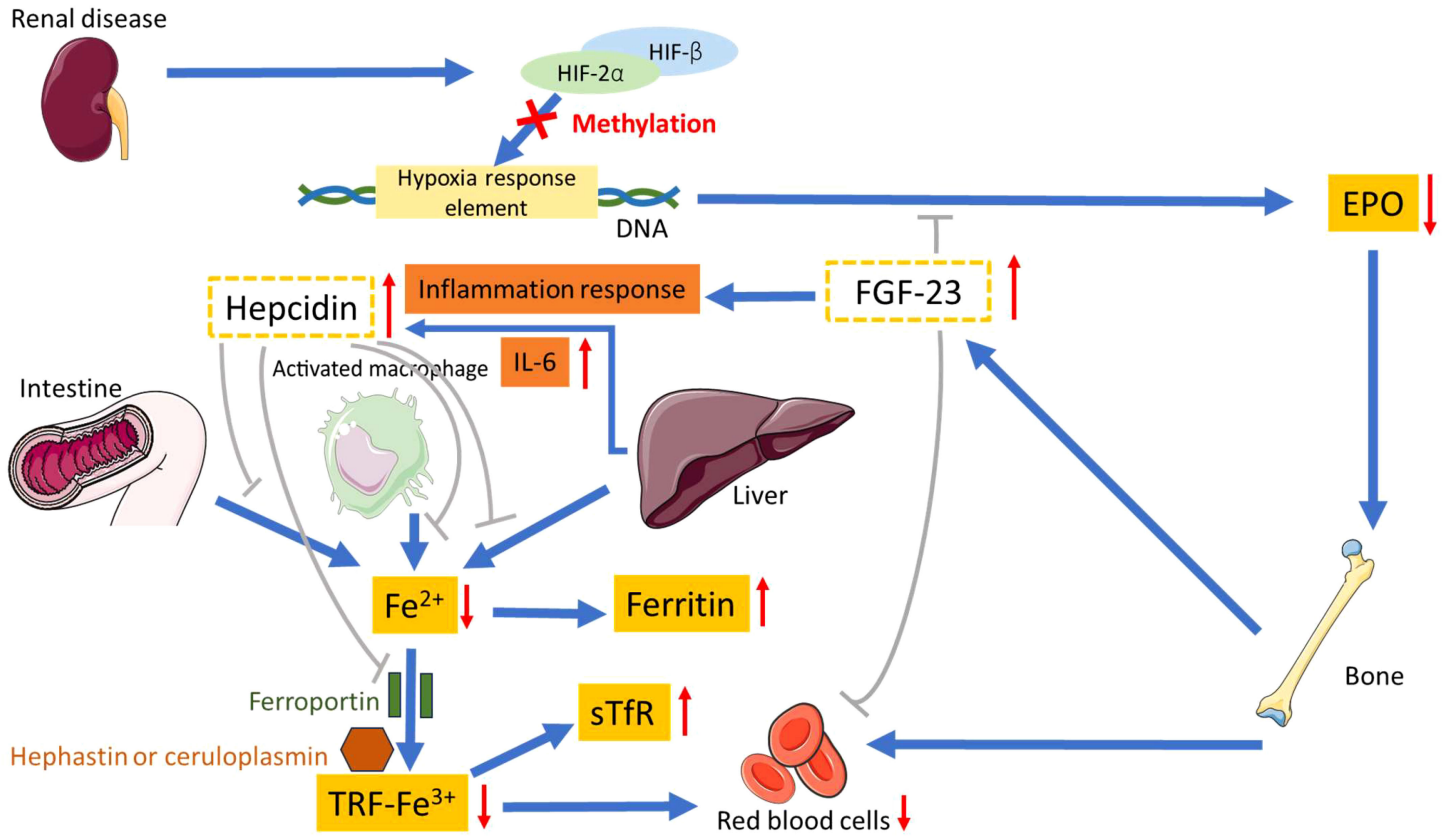
Proposed mechanism for renal anemia. HIF-2α, hypoxia-inducible factor-2α; HIF-β, hypoxia-inducible factor-β; EPO, erythropoietin; FGF-23, fibroblast growth factor 23; sTfR, soluble transferrin receptor; TRF, transferrin.

Multiple factors contribute to the exacerbation of anemia in CKD, with the principal factor being linked to erythropoietin (EPO) deficiency. Additional contributors to renal anemia encompass inflammation, iron deficiency, uremic inhibitors, a reduction in red blood cell survival, and vitamin B12 deficiency (14). The role of inflammation is increasingly acknowledged as a pivotal factor in renal anemia, complicating the diagnosis of either iron deficiency or a reduction in EPO renal production (15–18). Refer to **Figure 1** for a proposed mechanism elucidating renal anemia. In this scenario, renal disease prompts a reduction in EPO to inhibit bone-mediated RBC production. In CKD, bones excessively secrete the fibroblast growth factor 23 (FGF-23) hormone, capable of impeding EPO and RBC production, thereby contributing to renal anemia (19). In addition, FGF-23 elicits an inflammatory response and displays immunomodulatory properties (20). Numerous investigations provide evidence supporting the role of elevated FGF-23 as a causative factor in the initiation of renal anemia, chronic inflammation, and iron deficiency in the context of CKD (19, 21–23). The inflammation response in CKD, via FGF-23, promotes livers to increase interleukin-6 (IL-6) levels and induce hepcidin production (24–26). Increased hepcidin further inhibits intestinal iron absorption and iron release, leading to functional iron deficiency. This results in elevated expression of soluble transferrin receptor (sTfR), but the iron supply remains inadequate (27) The sTfR is the extracellular fragment derived from the cleavage of the cellular transferrin receptor. Diagnosis has progressed from traditional markers like ferritin to advanced tools such as sTfR and hepcidin, enhancing the detection of both absolute and functional iron deficiency anemia. The sTfR is not dependent on inflammation (28, 29). Consequently, the surge in hepcidin, binding to its cellular receptor ferroportin, obstructs macrophage iron release and intestinal iron absorption, leading to inhibited iron release from the liver and resulting in hypoferremia (30–32). Ferritin also increased linearly with increasing hepcidin (33).The overall effect of hepcidin is to curtail the availability of iron for active EPO, a phenomenon intricately linked to pathways involved in growth retardation in CKD (34–38). In renal disease, DNA relating to EPO becomes methylated, meaning that HIF cannot bind and promotes a decrease in EPO production. In addition, transferrin, an iron-transport glycoprotein, undergoes significant changes during acute phase responses in end-stage renal disease. In maintenance hemodialysis (mHD) patients, transferrin 2, transferrin 3, and transferrin 4 serum levels decrease, influenced by renal decline, prolonged mHD, and inflammation. These changes may contribute to persistent anemia (39).

**Figure 2:**
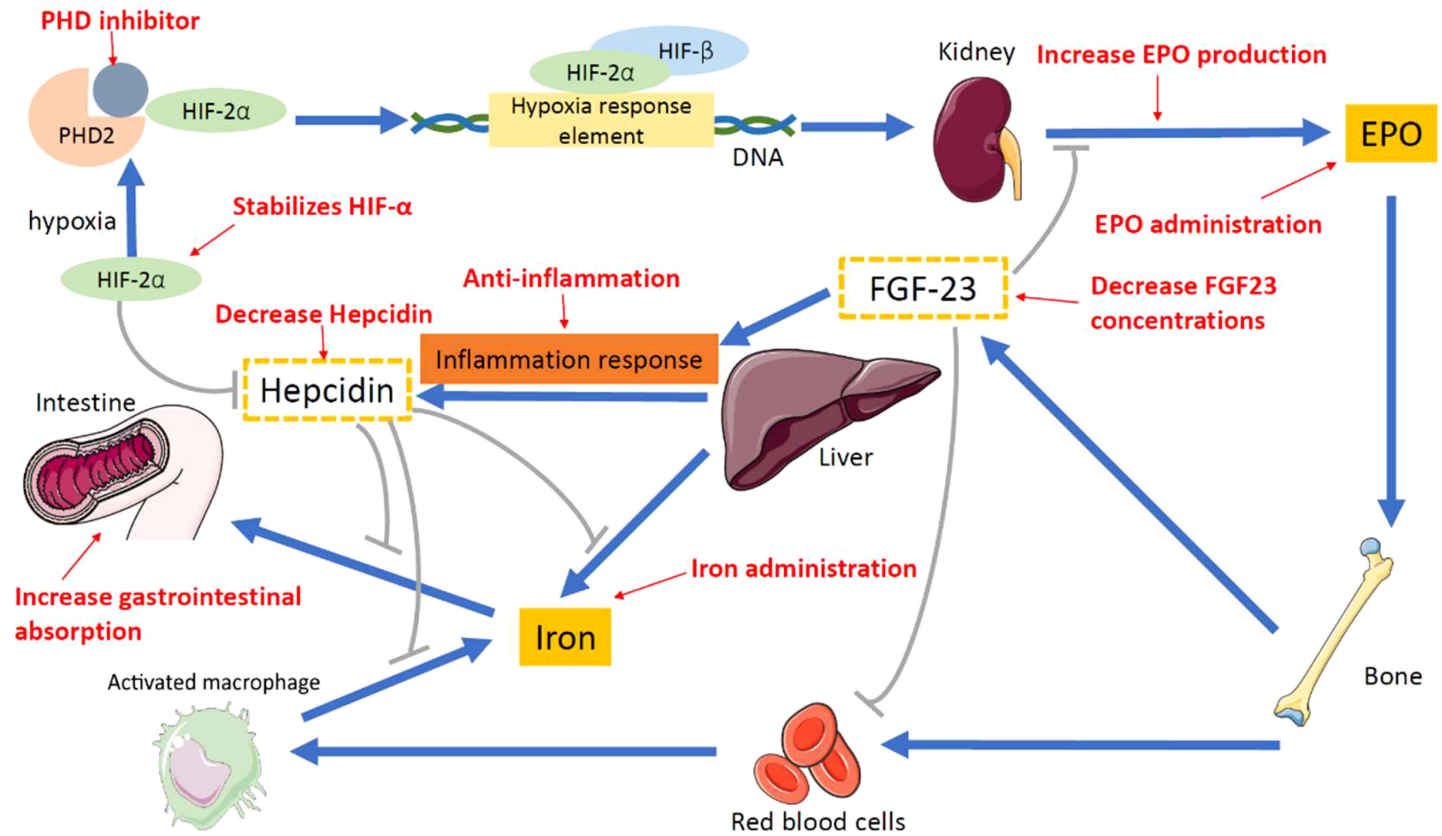
Proposed therapeutic methods for renal anemia. PHD inhibitor, prolyl hy-droxylase inhibitor; PHD, prolyl hydroxylase; HIF-2α, hypoxia-inducible factor-2α; HIF-β, hypoxia-inducible factor-β; EPO, erythropoietin; FGF-23, fibroblast growth factor 23.

**Figure 2** shows the therapeutic methods for renal anemia. Prolyl hydroxylase domain (PHD) oxygen sensors function as dioxygenases that modulate the activity of the hypoxia-inducible factor (HIF). This factor, in turn, governs the production of erythropoietin in both renal and hepatic contexts, orchestrating erythropoiesis in conjunction with iron metabolism (40–42). In hypoxic environments, the hydroxylation activity of PHDs experiences inhibition. This inhibition results in an elevation of the cellular concentration of HIF, leading to increased endogenous EPO production, enhanced iron absorption, and reduced levels of hepcidin. Consequently, the control of this pathway is commonly referred to as the HIF–PHD axis (42–44).

Reviews of conventional treatments for renal anemia have been published (32, 45). However, they often entail side effects (46), necessitating exploration of alternative adjunct therapies. Current evidence suggests that dietary control, nutritional supplements, and complementary and alternative medicine (CAM) can improve renal anemia (47, 48). A comprehensive review article on medications, dietary control, nutritional supplements, and CAM for renal anemia is currently lacking. Therefore, we conducted a narrative review to assess the effectiveness of these treatments in patients with renal anemia. Given the aforementioned mechanisms, this review delineates potential non-extracorporeal approaches applicable from laboratory research to clinical application, aiming to elevate hemoglobin (Hb) levels and enhance renal function. The search encompassed databases from their inception to 1 August 2023 including Embase, Cochrane Library, PubMed, Airiti Library, Wanfang, and Chinese National Knowledge Infrastructure, using the term “renal anemia.” To augment the search scope, we conducted further scrutiny of included articles and citations, utilizing the “related articles” feature on PubMed. The structure of this paper is as follows: The initial section concentrates on pharmaceutical interventions, encompassing HIF-PHIs, iron supplements, and EPO products. The subsequent section delves into dietary control and supplementary therapies, encompassing nutraceuticals prebiotics, and probiotics. The concluding section expounds on the utilization of CAMs and supplementary therapeutic modalities.

## 2 Conventional medication therapy

In standard medical interventions that are implemented to control any current underlying diseases, the Hb serum concentration is mainly raised by administering drugs to slow down kidney deterioration. This therapeutic strategy involves augmenting the inherent synthesis of erythrocytes, ensuring ample iron levels for hemoglobin formation, reducing cytokine production and release, implementing antioxidative and anti-inflammatory processes, suppressing hepcidin, and addressing anemia (49), overseeing and safeguarding renal EPO-producing cells (REPs) during stressful conditions for the treatment of renal anemia (50, 51), enhancing the production of endogenous EPO, optimizing iron utilization under hypoxia (52), inhibiting prolyl hydroxylases, and modulating hepcidin activity (53). Moreover, new studies have shown that the simultaneous correction of iron deficiency and hyperphosphatemia in CKD reduces the magnitude of FGF-23 increase. Thus, using iron-based phosphate binders in CKD might mitigate cardiac and renal injury and improve survival (54). In addition, dialysis improves hematocrit levels by reducing the plasma volume and increases RBC mass by removing middle molecule uremic toxins that affect RBC survival and EPO efficiency (55). Here is a concise overview of the existing treatment approaches that can be employed to elevate Hb levels (**Table 1**).

**Table 1:** Medications that can be used for renal anemia.

Intervention	Route, dosage, and frequency	Author/year	Mechanism/usage	Study design	Subjects	Subject number	Result
Clinical Studies							
Roxadustat	Oral, 100 mg or 70 mg, TIW	Chen N et al., 2019 (56)	Stabilizes HIF-2α subunits	Clinical trial	CKD stage 3–5 with no dialysis	101	Hb ↗
Vadadustat	Oral, 450 mg, QD	Pergola et al., 2016 (57)	Stabilizes HIF-2α subunits	Clinical trial	NDD-CKD stages 3a/b, 4, and 5	138	Reticulocytes ↗, plasma EPO ↗, and Hb ↗
Daprodustat	Oral, 5–25 mg, QD	Meadowcroft et al., 2019 (58)	Stabilizes HIF-2a subunits	RCT	HDD-CKD	177	Stabilizes Hb, plasma EPO ↗
Molidustat	Oral, 25–75 mg, QD	Macdougall et al., 2018 (59)	Stabilizes HIF-2a subunits	Clinical trial	NDD-CKD stages 3	121	Hb ↗
Ferric citrate	Oral, 2 g, TID	Womack et al., 2020 (60)	Decreases FGF-23	RCT	NDD-CKD stages 3b–4	30	TSAT ↗, SF ↗, SI ↗, and Hb ↗
Ferric maltol	Oral, 30 mg, BID	Pergola et al., 2021 (61)	Increases Fe^3+^ absorption in gastrointestinal tract	RCT	CKD stage 3 or 4 with iron-deficiency anemia	111	SI ↗ and Hb ↗
Liposomal iron	Oral, 30 mg, QD	Pisani et al., 2015 (62)	Increases Fe3+ absorption in gastrointestinal tract	RCT	CKD stage 3–5 with iron-deficiency anemia	66	Hb ↗
Ferumoxytol	IV, 1020 mg, single dose	Khan et al., 2021 (63)	Increases Fe3+ absorption in gastrointestinal tract	Clinical trial	CKD	140	Hb ↗
Ferric carboxymaltose	IV, 500–1000 mg, single dose	Macdougall et al., 2014 (64)	Maintains ferritin levels	RCT	NDD-CKD	155	Hb ↗
Iron isomaltoside	IV, 1000 mg, single dose	Bhandari et al., 2021 (65)	Quickens iron repletion	RCT	NDD-CKD	1027	Hb ↗
Ferric pyrophosphate citrate	IV, containing 2 µM FPC-iron	Fishbane et al., 2015 (66)	Directly donates iron to transferrin, by bypassing the reticuloendothelial system and avoiding iron sequestration	Clinical trial	HDD-CKD	299	Hb ↗ and iron ↗
Epodion	IV, 50 units/kg, TIW	Angginy et al., 2022 (67)	Biosimilar alpha rhEPO product	Clinical trial	HDD-CKD	200	1. Hb ↗ 2. Safe, with no immunogenetic reaction
Methoxy polyethylene glycol-epoetin beta	IV, 0.6 mg/kg, every 2 weeks	Locatelli et al., 2019 (68)	Alpha rhEPO attachment of a large methoxy polyethylene glycol polymer chain	RCT	CKD	640	1. Median Hb maintains 10–12 g/dL 2. Median SF ≥ 100 ng/m 3. Median TSAT ≥ 20%
Darbepoetin alfa (DA)	IV, maintain Hb levels within the target range of 10–12 g/dL over 24 weeks	Ohki et al., 2020 (69)	Increases RBC mass and viscosity	Clinical trial	NDD-CKD	18	Maintains Hb levels but requires long-acting ESAs

↗, increase; HIF, hypoxia-inducible factor; HIF-PH inhibitor, hypoxia-inducible factor prolyl hydroxylase inhibitor; HDD-CKD, hemodialysis-dependent chronic kidney disease; NDD-CKD, non-dialysis-dependent chronic kidney disease; EPO, erythropoietin; rhEPO, recombinant human erythropoietin; FGF-23, fibroblast growth factor 23; SF, serum ferritin; TSAT, transferrin saturation; Hb, hemoglobin; HCT, hematocrit; SI, serum iron; HD, hemodialysis; CKD, chronic kidney disease; i.v., intravenous; TID, ter in die; QD, quaque die; BID, bis in die; TIW, three times a week.

### 2.1 Roxadustat

By orally inhibiting hypoxia inducible factor prolyl hydroxylase (HIF-PH), Roxadustat induces erythropoiesis and modulates iron metabolism. This is achieved through the reduction of serum hepcidin levels and an increase in the absorption of iron in the intestine. Moreover, the increased levels of endogenous erythropoietin further increase hemoglobin levels and improve iron homeostasis (70–72). Additionally, Roxadustat exhibits the potential to enhance renal osteodystrophy (ROD) by concurrently handling bone remodeling. Moreover, the use of Roxadustat represents a potentially promising approach in the treatment of osteoporosis (73). During an 8week clinical trial, Roxadustat treatment was administered to 101 non-dialysis patients with stages 3–5 CKD. Subsequently, a notable reduction in both hepcidin and cholesterol levels was observed, leading to a significant elevation in hemoglobin levels (56).

### 2.2 Vadadustat

Vadadustat, an innovative, adjustable, orally administered inhibitor of hypoxia inducible factor prolyl hydroxylase, can activate HIF signaling and induce endogenous erythropoietin synthesis, which stimulates iron mobilization and inhibits FGF-23 (74, 75). Vadadustat treatment in animal studies has ameliorated anemia and also decreased levels of serum urea nitrogen and creatinine concentrations alongside the appearance of kidney fibrosis markers (76). In a randomized clinical trial spanning 20 weeks, 138 non-dialysis patients with stages 3–5 CKD were subjected to Vadadustat treatment. Consistently, their hemoglobin levels showed an increase and were sustained, accompanied by heightened iron mobilization as evidenced by a significant rise in both reticulocytes and total iron binding. Moreover, Vadadustat treatment led to a notable reduction in both serum hepcidin and ferritin levels (57).

### 2.3 Daprodustat

Daprodustat is an oral HIF-PH enzyme that inhibits PHD1, PHD2, and PHD3, which stimulates erythropoiesis. Meanwhile, it has the capacity to boost endogenous EPO production by stabilizing the HIF-α subunit, facilitating its dimerization with the HIF-β subunit, and activating target genes crucial for protecting against hypoxia, such as the erythropoietin gene (77). In a randomized clinical trial, 177 patients with HD switched from being treated with rhEPO to Daprodustat for 24 weeks, which worked to maintain and stabilize their Hb levels (58).

### 2.4 Molidustat

Molidustat, a orally bioavailable inhibitor of HIF-PH, emulates hypoxia by stabilizing the HIF-α subunits. It orchestrates a physiological response by triggering the transcription of erythropoietin and hypoxia inducible genes, encompassing those linked to erythropoiesis, angiogenesis, and mitochondrial metabolism (78). Following a 16week randomized clinical trial involving 121 dialysis patients, treatment with Molidustat resulted in a significant elevation of their hemoglobin levels (59, 79). Hence, Molidustat emerges as an effective and generally well-tolerated substitute for darbepoetin in the management of renal anemia.

### 2.5 Ferric citrate

Ferric citrate (FC) serves as an oral, calcium-free, iron-based phosphate binder, effectively lowering serum phosphorus levels by inhibiting phosphate absorption in the gastrointestinal tract and concurrently addressing anemia through iron supplementation (80). In addition, FC and HIF-PHIs have been shown to significantly decrease FGF-23 levels and renal anemia (81, 82). Moreover, numerous studies have indicated that ferric citrate may reduce circulating FGF-23 levels, potentially achieved through the modulation of dietary phosphate absorption (60, 83, 84). In a randomized clinical trial, 30 patients were treated with ferric citrate for 12 weeks, and their TSAT, ferritin, iron, and Hb were significantly increased (60).

### 2.6 Ferric maltol

Ferric maltol, a compound comprising ferric iron and maltol, a naturally occurring sugar derivative, demonstrates stability at a physiological environment. This complex remains securely chelated in the intestinal lumen until absorption, where the iron transport receptor on luminal enterocytes facilitates dissociation from maltol. Consequently, the absence of free iron in the gut diminishes the generation of hydroxyl radicals, minimizing the risk of gastrointestinal toxicity. In a 16week randomized clinical trial involving 111 patients, ferric maltol treatment resulted in significant increases in hemoglobin, ferritin, transferrin saturation, and serum iron levels. Notably, this treatment was well-tolerated for up to 1 year (61, 85).

### 2.7 Liposomal iron

Liposomal iron, a formulation of ferric pyrophosphate encapsulated within phospholipids and sucrose esters derived from the fatty acid membrane, represents an advanced generation of oral iron characterized by its superior gastrointestinal absorption, high bioavailability, and minimal incidence of side effects (62, 86). Utilizing advanced liposome-based technology as a carrier, this innovative approach ensures that iron bypasses direct contact with the gastrointestinal mucosa. Instead, absorption occurs directly in the intestine. Within the intestinal lumen, M cells in the small intestine, originating from the lymphatic system, directly absorb the liposome. Following this, macrophages incorporate the liposome intact through endocytosis into the lymphatic system, enabling it to reach hepatocytes (87). Then, lysosomal enzymes facilitate the opening of the liposome, leading to the release of iron. In a randomized clinical trial, 66 patients received oral liposomal iron for 3 months, and their Hb levels increased significantly, while the number of adverse events also decreased significantly (62).

### 2.8 Ferumoxytol

Ferumoxytol is a formulation of iron that is delivered through an IV. There are only a limited number of gastrointestinal side effects from ferumoxytol treatment. However, its efficacy is notably enhanced in individuals with malabsorption syndromes or those who have undergone gastric surgery. Yet, it is important to note the potential for adverse events, which may encompass hypersensitivity reactions and anaphylactic shock in extremely rare instances (46, 63). In a clinical trial, 140 patients received IV ferumoxytol infusions and their Hb levels increased significantly and safety (63).

### 2.9 Ferric carboxymaltose

The utilization of intravenous iron treatments facilitates the prompt correction of iron-deficiency anemia. However, in comparison to iron isomaltoside, the use of ferric carboxymaltose resulted in a higher incidence of hypophosphatemia, which was mediated by fibroblast growth factor 23 (88, 89). In a randomized clinical trial involving 155 patients in the high ferritin group, a rapid and sustained achievement of consistent hemoglobin levels was observed. This outcome led to the delayed or reduced necessity for other anemia management interventions, such as erythropoiesis stimulating agents (ESAs), with no observed renal toxicity and no discernible difference in cardiovascular or infectious events (64).

### 2.10 Iron isomaltoside

The recommendation for high-dose, low-frequency intravenous iron treatments aims to potentially enhance symptoms, functional capacity, and overall quality of life for patients. In a randomized clinical trial involving 1027 individuals diagnosed with non-dialysis-dependent CKD, the administration of a single dose of iron isomaltoside resulted in a faster and transiently greater hemoglobin response compared to the treatment with multiple doses of iron sucrose. Additionally, lower rates of hypersensitivity reactions were observed, accompanied by a significant decrease in the incidence of composite cardiovascular diseases (65).

### 2.11 Ferric pyrophosphate citrate

Ferric pyrophosphate citrate (FPC) stands out as a carbohydrate-free, water-soluble, complex iron salt capable of delivering iron through dialysate, effectively sustaining hemoglobin concentrations and iron balance. This innovative approach has demonstrated the potential to reduce the requirement for intravenous (IV) iron by approximately 80% (90). FPC exhibits the capability to traverse the dialyzer membrane, entering the bloodstream to directly contribute its iron to transferrin before undergoing rapid clearance. Moreover, FPC serves as a source of iron for erythropoiesis, circumventing iron sequestration within reticuloendothelial macrophages and hepcidin induced iron entrapment (90, 91). In a randomized clinical trial, which included 299 chronic hemodialysis patients, dialysate containing 2 μM FPC-iron was administered for 48 weeks. The Hb levels in the patients receiving the treatment increased and were maintained. Concurrently, FPC demonstrated a lack of increase in iron stores among the patients and exhibited a safe profile, suggesting its potential for future use (66).

### 2.12 Epodion

Epodion, an alpha rhEPO, is a biosimilar exogenous EPO product. Epodion is a yellowish, transparent solution that can be injected intravenously (IV) or subcutaneously (SC). Moreover, in a clinical trial with 200 patients, epodion was administered for 52 weeks and resulted in an increase in Hb levels without demonstrating any adverse immunogenetic reaction (67, 92).

### 2.13 Methoxy polyethylene glycol-epoetin beta

Methoxy polyethylene glycol epoetin beta, an attachment to an extensive methoxy polyethylene glycol polymer chain, represents a modified form of recombinant human erythropoietin epoetin. The introduction of these modifications has elevated the treatment’s efficacy, leading to less frequent administration and subsequently reducing the treatment burden on both patients and healthcare providers. However, the potential increase in cardiovascular risk associated with erythropoiesis stimulating agents prompted a randomized clinical trial involving 640 patients treated with methoxy polyethylene glycol epoetin beta compared to 644 patients receiving shorter acting epoetin alfa/beta agents, with both treatments administered to target hemoglobin levels of 10–12 g/dL. The trial results indicated that the once monthly methoxy polyethylene glycol epoetin beta treatment was non inferior to conventional, shorter acting erythropoiesis stimulating agents when assessing adverse cardiovascular events and all cause mortality (68).

### 2.14 Darbepoetin alfa

Developed specifically for the management of anemia associated with chronic kidney disease, darbepoetin alfa represents a novel erythropoiesis stimulating protein (93). However, the utilization of erythropoietin stimulating agents (ESAs) may be associated with elevated blood pressure (BP), inflammation, hyperparathyroidism, and malnutrition (94). In a clinical trial, 18 patients were treated with darbepoetin alfa (DA) for 24 weeks and their Hb levels were maintained at 10–12 g/dL, while the office/ambulatory BP profiles also did not decline (69).

## 3 Diet control and supplements

Renal anemia is a consequence of end-stage renal disease (ESRD). Contemporary investigations indicate a significant association between gut microbiota and the onset and progression of ESRD. Furthermore, there is a robust correlation between the gut microbiome and EPO hyporesponsiveness (EH) (95). Therefore, the modulation of gut microbiota is anticipated to represent an innovative therapeutic approach for CKD patients experiencing clinical refractory anemia. Furthermore, this intervention has the potential to decrease reliance on conventional ESA and iron agent medications, consequently mitigating the adverse effects associated with these drugs and enhancing the prognosis for individuals with kidney failure. The ensuing treatment strategies are succinctly examined below (**Table 2**).

**Table 2:** Dietary treatments for controlling renal anemia.

Intervention	Route, dosage, and frequency	Author/year	Mechanism/usage	Study design	Subjects	Subject number	Result
Clinical Studies							
Dietary fiber	Oral, 10 g/day for 8 weeks	Li Y et al., 2022 (95)	Modulated prebiotic activity and SCFAs	RCT	Patients undergoing HD	162	Hb ↗, Fe2+ ↗, SF ↗, *Lactobacillu* ↗, and *Lactobacillaceae* ↗
Probiotic capsule	Oral, 500 mg/day for 3 months	Zahra Shariaty et al., 2017 (96)	Probiotics established a balance between pro− and anti−inflammatory cytokines	RCT	Patients undergoing HD	36	Hb ↗
Probiotic capsule	Oral, 1 capsule/day for 3 months.	de Araújo ÉMR et al., 2022 (47)	Probiotics decreased systemic inflammation	RCT	Patients undergoing HD	32	Hb ↗

↗, increase; SCFAs, short-chain fatty acids; HIF, hypoxia-inducible factor; PHD, prolyl hydroxylase inhibitor; NDD, non-dialysis-dependent; EPO, erythropoietin; SF, serum ferritin; Hb, hemoglobin; HD, hemodialysis.

### 3.1 Dietary fiber

Dietary fiber (DF), primarily obtained from plant-based foods, is a polysaccharide that undergoes limited digestion in the gastrointestinal tract and is instead utilized by the gut microbiota (97, 98). Nevertheless, certain bacteria present in the human gut have the capability to utilize dietary fiber (DF) as a catabolic substrate, leading to the production of substances like bile acids and short chain fatty acids (SCFAs). These compounds play a crucial role in modulating inflammation and oxidative stress. Consequently, SCFAs contribute to the overall well-being of the microbiome and mucosa, offering a range of health benefits, including antidiabetic, anticancer, antibacterial, anti-inflammatory, and antioxidative effects (99). Research findings indicate that elevated levels of short chain fatty acids (SCFAs) in feces and/or serum have been associated with alleviation of renal anemia (100, 101). Furthermore, additional studies have demonstrated that the presence of Bifidobacterium adolescentis, Lactobacillus, and Lactobacillaceae increased in the dietary fiber (DF) group. Interestingly, Lactobacillus and Lactobacillaceae were found to be positively correlated with hemoglobin (Hb) and Fe2+ levels, and inversely correlated with recombinant human erythropoietin (rhEPO) dosage (95). Therefore, the regulation of gut microbiota and modulation of short chain fatty acids (SCFAs) by dietary fiber (DF) present a potential avenue for enhancing renal anemia in individuals with end stage renal disease (ESRD).

### 3.2 Probiotic capsule

Probiotics play a role in establishing a balance between pro- and anti-inflammatory cytokines, which could potentially be linked to renal anemia. Studies have shown that probiotic supplementation can decrease Hb fluctuations in hemodialysis patients; however, no significant increase was observed in the Hb level (96). Further, in a randomized clinical trial, 32 patients were provided with oral probiotic supplements for 3 months. Thereafter, a notable reduction was observed in both syndecan-1 and blood glucose levels, suggesting potential enhancements in metabolism and a decrease in systemic inflammation (47).

## 4 Complementary and alternative medicinal therapies

CAM therapy finds extensive application in addressing CKD with renal anemia. Mounting evidence indicates that CAM therapy holds the potential to ameliorate renal anemia by mechanisms including enhanced iron metabolism, anti-inflammatory effects, diminished hepcidin levels, and heightened expressions of EPO and HIF (102, 103). Hence, this section provides a concise overview of the positive impact of CAM therapies on CKD with renal anemia. The CAM therapeutic modalities employed for CKD encompass traditional Chinese medicine (TCM) decoction, TCM monomers, herbal monomers, acupoint application, acupoint injection, and ginger moxibustion (**Table 3**).

**Table 3:** CAM treatments being used to control renal anemia.

Intervention	Route, dosage, and frequency	Author/year	Mechanism/usage	Study design	Subjects	Subject number	Result
Clinical Studies							
Shengxuening tablets	Oral, 1.0 g, TID	Tang et al., 2020 (104)	Improved the microinflammatory state	RCT	HDD-CKD	94	Fe2+ ↗, SF ↗, TSAT ↗, and Hb ↗
Shengxuening tablets	Oral, 1.0 g, TID	Cheng et al., 2016 (105)	Improved iron metabolism, increased serum iron and transferrin saturation and promoted hematopoiesis	RCT	HDD-CKD	72	Hb ↗, HCT ↗, SF ↗, and transferrin ↗
Shengxuening tablets	Oral, 0.5g, TID	Lin et al., 2016 (106)	Supplemented iron	RCT	Patients with stages 3~4 CKD	96	Hb ↗, SF ↗, and TSAT ↗
Qingshen granules	Oral, 0.3g, TID	Zhang et al., 2019 (107)	Improved iron metabolism through anti-inflammation, and reduced hepcidin levels	RCT	NDD-CKD	60	Hb ↗, HCT ↗, RBC ↗, SI ↗ TAST ↗, IL-6 ↘, hs-CRP ↘, and Hep ↘
Danggui Buxue decoction	Oral, 200 mL, QD	Lu et al., 2022 (108)	Anti-inflammation	RCT	HDD-CKD	110	Hb ↗, RBC ↗, and HCT ↗
Danggui Buxue decoction	Oral, 1 dose, QD	Zhao et al., 2017 (48)	Anti-inflammation and promoted hematopoiesis	Meta	Renal anemia with CKD	460	Hb ↗, RBC ↗, and HCT ↗
Zishen Shengxue recipe	Oral, 1 capsule, QD	Wu et al., 2022 (109)	Inhibited Hep, which promoted the production of RBCs	RCT	HDD-CKD	80	Hb ↗, HCT ↗, SF ↗, TSAT ↗, and Hep ↘
Jianpi Shengxue tablets	Oral, 1.8 g, TID	Jian et al., 2018 (110)	Regulated the secretion of hepcidin	RCT	Stage 3 NDD-CKD	60	Hb ↗, RBC ↗ Hct ↗, SI ↗, and SF ↗
Jiawei Shiquandabu decoction	Oral, 1 dose, QD	Yao et al., 2016 (111)	Cleared oxygen-free radicals to protect RBCs	RCT	HDD-CKD	120	Hb ↗ and Hct ↗
Yishen Jiangzhuo decoction	Oral, 1 dose, QD	Ren et al.,2021 (112)	Anti-inflammation and upregulated HIF expression	RCT	Stages 3–4 CKD	80	RBC ↗, Hb ↗, Hct ↗, and hs-CRP↘
Acupoint application	Application of drugs, 10–16 h/d	Shi et al., 2022 (113)	Improved anemia to protect renal cells	RCT	HDD-CKD	100	Hb ↗ and Hct ↗
Wenshen Jiedu ointment	Cream, QD	Zhu, 2020 (114)	Promoted EPO production	Clinical trial	Patients with renal anemia	100	Hb ↗ and Hct ↗
EPO acupoint injection	i.m., QW	Cheng, 2017 (115)	Reduced inflammation and improved EPO resistance	RCT	HDD-CKD	200	Hb ↗ and Hct ↗
EPO injection and Houttuynia cordata injection	i.m., 1mL/point, QOD	Xiong et al., 2006 (116)	Reduced inflammation	RCT	NDD-CKD	43	Hb ↗
Astragalus acupoint injection	i.m., 0.3–0.5mL/point, TIW	Tan, 2015 (117)	Promoted EPO production	RCT	Stage 4 CKD	120	Hb ↗ and Hct ↗
Ginger moxibustion	Ginger (diameter: 4–5 cm, thickness: 0.2–0.3 cm), QOD	Liu et al., 2021 (118)	Reduced inflammation	RCT	Stages 2–4 NDD-CKD	80	Hb ↗
Animal Studies							
Siwu granules	Gastric gavage, 2.3 g/kg, QD	Wu et al., 2019 (119)	Antioxidant and anti-inflammation	Animal	Adenine-induced renal injury rats	50	Hb ↗
Jian-Pi-Yi-Shen	Intraperitoneally injected, 6.0 g/kg, QD	Wang et al., 2020 (103)	Induced EPO production, regulated iron metabolic targets, and translational control of HIF-2α protein	Animal	5/6 nephrectomy-induced CKD rats	48	RBC ↗, HGB ↗, and Hct ↗
Steamed Panax notoginseng	Gastric gavage, 1.8 g/kg, QD	Gao et al., 2022 (120)	Restored EPO mRNA expression in the kidneys and EPO receptor mRNA in bone marrow nucleated cells	Animal	Adenine-induced renal anemia injury rats	70	RBC ↗
Luteolin	Gastric gavage, 100 mg/kg	Siyu et al., 2020 (121)	Inhibited PHD2/HIF-2α axis and oxidative stress in kidney	Animal	HgCl2-induced renal anemia rats	28	Hb ↗
Jujube polysaccharides (JP)	Gastric gavage, 1.2 g/kg/d	Huang, Shiying et al., 2021 (122)	Alternated the EPO level via HIF-α signaling	Animal	Nephrectomy rats	12	Hb ↗

↗, increase; , decrease; SF, serum ferritin; TSAT, transferrin saturation; Hb, hemoglobin; HCT, hematocrit; SI, serum iron; IL-6, interleukin-6; hs-CRP, hypersensitive C-reactive protein; Hep, hepcidin; HD, hemodialysis; HDD-CKD, hemodialysis-dependent chronic kidney disease; NDD-CKD, non-dialysis-dependent chronic kidney disease; i.m., intramuscular injection; TID, ter in die; QD, quaque die; QW, every week; QOD, quaque omni die; TIW, three times a week.

### 4.1 Shengxuening tablet

Shengxuening tablets (SXN) are an extract from silkworm excrement. The main component is chlorophyll and its derivatives, and they have a similar structure to heme, making them an effective biological iron supplement. Moreover, SXN can enhance the uptake of free iron and stimulate bone marrow cell proliferation. In a recent clinical trial, 94 patients with renal anemia undergoing mHD were randomly assigned to either the SXN group (receiving oral SXN tablets) or the FS group (oral treatment with ferrous succinate tablets). In both groups, Hb and transferrin saturation (TSAT) levels demonstrated a significant rise compared to the screening period. However, no notable distinction was observed between the two treatment groups. Nevertheless, the amount of EPO administered in the SXN group was less than that in the FS group, suggesting that the administration of SXN tablets can lead to a decrease in the utilization of EPO and effectively ameliorate renal anemia (104, 123). Furthermore, another recent clinical trial reported that SXN tablets combined with rHuEPO could increase Hb, TSAT, SF, and Hct levels and decrease the consumption of rHuEPO compared to the control group (105). Additionally, Lin et al. demonstrated that SXN tablets could improve iron metabolism and were a safe and effective treatment option, with a reduced dosage of EPO, for renal anemia in patients with stages 3–4 CKD (106).

### 4.2 Qingshen granules

Qingshen granules (QG) are a preparation used in TCM that has been clinically demonstrated to delay the progression of renal fibrosis in patients with CKD. Earlier investigations have also substantiated that QG can ameliorate the inflammatory condition in patients with CKD by decreasing the serum levels of IL-6, TNF-α, and hs-CRP, as well as mitigating renal fibrosis in patients concurrently experiencing chronic renal failure (123–126). In a preceding clinical study, disclosed in 2019, investigators examined the physiological data of 60 patients with CKD in stages 3–5. These participants were arbitrarily allocated into a QG group and a control group. After treatment for 12 weeks, the levels of HGB, HCT, RBC, SF, and TSAT had increased in both groups, and the levels of hs-CRP, IL-6, and hepcidin decreased; however, the improvements were more apparent within the QG group. The results indicated that QG can effectively improve renal anemia in CKD patients, potentially by enhancing iron metabolism through mitigating inflammation and reducing hepcidin levels (107).

### 4.3 Danggui Buxue decoction

Danggui Buxue decoction (DBD) is another TCM preparation. DBD is composed of Angelica and Astragalus and is often used to treat coronary heart disease and anemia. A study showed that DBD has Quercetin and can modulate multiple inflammatory proteins and pathways in response to renal anemia (127, 128). A recent clinical trial, which included 110 patients with renal anemia and dialysis-dependent CKD, showed a notable rise in RBCs, Hb, and HCT levels following treatment with DBD combined with L-carnitine for 3 months, compared to the control group. These results confirmed that DBD in conjunction with L-carnitine can improve anemia-related symptoms and renal function by reducing the damage of inflammatory mediators to renal tissue (108). A meta-analysis of seven studies revealed that DBD combined with conventional Western medicine (CWM) was more effective and safe than CWM alone (48).

### 4.4 Zishen Shengxue recipe

In China, a Zishen Shengxue recipe (ZSR) consisting of eight herbs is often used to treat renal anemia with CKD. In a recent clinical study, 80 participants were enrolled and randomly allocated into two groups: EPO subcutaneous injection and EPO subcutaneous injection plus ZSR. Following 8 weeks of intervention, the ZSR group demonstrated a substantial elevation in the levels of Hb, HCT, and TSAT, in contrast to the control group, with a simultaneous significant reduction in Hep levels. Furthermore, a study by Wu et al. showed that ZSR combined with EPO had a definite clinically curative effect on patients with renal anemia, which was more advantageous than using EPO alone. A possible mechanism to explain this intervention is that Hep corrects the disorder of iron metabolism in the body and promotes the production of RBC (109).

### 4.5 Jianpi Shengxue tablets

Jianpi Shengxue tablets (JSTs) are a compound preparation of traditional Chinese and Western medicine, commonly used to treat anemia. Western medicine includes ferrous sulfate and vitamin C, while the TCM prescription includes common blood-enriching herbs, such as Codonopsis, Poria, Atractylodes macrocephala, licorice, astragalus, and gallinacean (129). Recently, a clinical trial compared the curative effects of JST and polysaccharide-iron complex capsules in the management of renal anemia without dialysis and found that the levels of Hb, RBCs, and HCT in the JST group showed a significant increase compared to the control group (110).

### 4.6 Jiawei Shiquandabu decoction

Jiawei Shiquandabu decoction (JSD) is a TCM preparation consisting of rhubarb, chuanxiong, ginseng, paeoniae alba, Atractylodes macrocephala, Angelica, Astragalus, cinnamon, licorice, Rehmannia and Poria. Previously, a clinical trial using JSD showed that administering JSD combined with EPO therapy for 3 months could increase the Hb and Hct levels in HD patients with renal anemia (111, 130).

### 4.7 Yishen Jiangzhuo decoction

Yishen Jiangzhuo decoction (YJD) is a TCM preparation that is composed of Pseudostellaria, Atractylodes macrocephala, Astragalus, Poria cocos, rhubarb, tangerine peel, angelica, mulberry, salvia, achyranthes bidentata, plantain seed, Serissa japonica, mulberry, and motherwort. YJD was shown to reduce Scr, BUN, and CRP levels, and increase RBC, Hb, and HCT levels. Moreover, the herbs in YJD, such as astragalus, angelica, and ginseng have been shown to stimulate bone marrow hematopoiesis and participate in erythropoiesis, possibly by upregulating HIF expression, activating downstream signaling, and promoting erythropoiesis (112).

### 4.8 Acupoint application

Acupoint application is an external treatment method in TCM, which can be used to determine the effect of medicine and acupuncture point stimulation at the same time. A recent clinical trial with 100 patients with CKD used EPO subcutaneous injections plus acupoint application (the medicinal ingredients: Astragalus, Eucommia, Dipsacus, raw rhubarb, Angelica, motherwort, Chuanqiong, raw oysters, Radix Aconiti) at Zusanli (ST36), Geshu (BI17), Pishu (BI20), and Shenshu (BI23) for 2 months; the patients demonstrated an increase in Hb and Hct levels in the acupoint application group, surpassing those in the control group (113). In addition, acupoint application is also effective in reducing BUN and serum creatinine levels (131). In a different clinical study, 100 participants were enrolled and randomly assigned to two groups: EPO subcutaneous injection and EPO subcutaneous injection plus Wenshen Jiedu ointment (the medicinal ingredients: Aconite, Rhizoma Chuanxiong, cinnamon, Asarum, Paeoniae alba, fairy pill, rhubarb, Pinellia, agarwood) acupoint application at Zusanli (ST36) and Shenshu (BI23). Zhu et al. indicated that Hb and Hct were significantly improved after treatment with Wenshen Jiedu ointment before and after treatment and between groups (114).

### 4.9 EPO acupoint injection (Zusanli)

Acupoint injection therapy injects drugs into acupoints, stimulates acupoints and meridians through acupuncture and medicinal liquid, and integrates meridians, acupoints, and drug effects. It represents a successful example of the clinical utilization of combined traditional Chinese and Western medicine (117, 132). A clinical trial by Cheng et al. found that the EPO Zusanli acupoint injection improved EPO resistance and enhanced the efficacy of EPO by alleviating the microinflammatory state of the body (115).

### 4.10 EPO injection and Houttuynia cordata injection (Shenshu and Zusanli)

Houttuynia cordata is a TCM herb that is often used to prevent and treat colds and has been clinically proven to have anti-inflammatory effects. In a preliminary investigation released in 2006, scientists examined physiological data from 43 non-dialysis CKD patients. These individuals were arbitrarily allocated into two groups: EPO subcutaneous injection plus Houttuynia cordata Shenshu (BI23) and Zusanli (ST36) acupoint injection, and EPO subcutaneous injection. The results indicated a more significant increase in Hb after acupoint injection with Houttuynia cordata (116).

### 4.11 Astragalus acupoint injection (Shenshu, Zusanli, Sanyinjiao, and Pishu)

Modern pharmacological studies have shown that Astragalus has a similar effect to erythropoietin, and can promote the production, development, and maturation of various blood cells. Furthermore, a clinical trial, which included patients with stage 4 CKD, revealed that treatment with EPO subcutaneous injection plus astragalus Shenshu(BI23), Zusanli(ST36), Sanyinjiao(SP6), Pishu(BI20) acupoint injection facilitated a notable rise in Hb, Hc, and Ret levels, in contrast to the EPO subcutaneous injection group. Tan et al. indicated that Astragalus acupoint injections could yield a therapeutic effect on stage 4 renal anemia in CKD and diminish the usage of EPO, although the specific mechanism of action needs further study (117).

### 4.12 Ginger moxibustion (Shenque, Guanyuan, and Zusanli)

Moxibustion is a CAM therapy involving the combustion of dried moxa at specific acupoints on the body. The principle of the moxibustion effect is to induce a warming effect alongside radiation and pharmacology effects (130). Ginger moxibustion is a kind of indirect moxibustion, which achieves a therapeutic effect through the dual warming and medicinal effects of moxa and ginger. A clinical trial involving 80 patients with CKD found that ginger moxibustion therapy led to a significant decrease in BUN, serum CRP, and creatinine levels, while inducing Hb levels. Moreover, Liu et al. indicated that ginger moxibustion has the potential to markedly enhance the inflammatory condition in CKD patients, increase Hb levels, improve renal function, and reduce disease progression (118).

### 4.13 Siwu granules

Siwu granules, encompassing Angelica, Ligusticum, and Rehmannia glutinosa, are employed in TCM and constitute a classical formula for promoting blood circulation. Wu et al. found that in rats with adenine-induced renal injury, the administration of Siwu granules in conjunction with EPO treatment elevated the expression of EPO and EPOR in renal tissues by enhancing the expression of endogenous EPO or mitigating EPO resistance. Furthermore, it was observed that in the Siwu plus EPO group, oxidative stress and inflammatory factors were inhibited, resulting in improved renal function and anemia (119).

### 4.14 Jian-Pi-Yi-Shen

Jian-Pi-Yi-Shen (JPYS) is a TCM formulation comprising eight herbs, often utilized in the management of CKD and associated complications, including anemia. JPYS enhanced red blood cells (RBCs), hemoglobin (HGB), and hematocrit (HCT) levels by triggering the expressions of EPO and hypoxia-inducible factor-2 alpha (HIF-2α) proteins in rats with anemia induced by 5/6 nephrectomy (103).

### 4.15 Steamed Panax notoginseng

Panax notoginseng (PN) is a TCM herb, which is available in two forms. Moreover, it is frequently employed to address hematological issues, with unprocessed PN utilized for inflammation and pain treatment, and processed steamed PN (SPN) serving as a “blood-enriching” remedy to alleviate anemia and boost overall immunity. A study on rats with adenine-induced renal anemia suggested that treatment with SPN could alleviate renal anemia by restoring the expression of EPO mRNA in the kidneys and EPO receptor mRNA in bone marrow nucleated cells (120).

### 4.16 Luteolin

Luteolin (Lut) is a natural flavonoid found ubiquitously in the diet and possessing numerous biological activities (133). A group of HgCl2 mice displaying anemia were treated with Lut, which could alleviate the anemia. Further studies have shown that Lut inhibits PHD2 in the kidney, a finding supported by a molecular docking study, and reinstates the expression of downstream proteins of PHD2, namely HIF-2α and erythropoietin. Additionally, Lut alleviates renal oxidative stress by enhancing the expression of antioxidant enzymes downstream of HIF-2α. To sum up, Lut alleviates renal anemia in mice by blocking the PHD2/HIF-2α axis and mitigating oxidative stress (121).

### 4.17 Jujube polysaccharides

Jujube polysaccharides (JPs) represent a category of active dietary glycans present in the fruit of Ziziphus jujuba. The application of jujube extract yielded positive outcomes, including the modulation of EPO through the activation of hypoxia-inducible factor (HIF) induced erythropoietin, the potential capability to recycle heme iron during erythrophagocytosis, and the bidirectional regulation of the immune response (134).Therefore, the suggested function of jujube in nourishing the blood is being proposed (135). A previous study has shown in a CKD rat model that treatment with JP substantially improved renal function and mitigated kidney pathological damage, while elevating RBC, Hb, hematocrit, and platelet counts (122). Additionally, JP stimulated the release of short-chain fatty acids (SCFAs) in rats with CKD, along with modulating the levels of kidney EPO mRNA and kidney EPO protein through HIF-α signaling.

## 5 Study limitations

Elevated ferritin levels and reduced iron saturation were associated with lower hemoglobin levels, with ferritin increasing further in advanced CKD stages. Anemia management in CKD varies by disease stage and dialysis status, requiring a tailored approach (136). Evaluating iron metabolism in CKD is intricate and should be individualized to ensure optimal care.

## 6 Conclusions

Various interventions, encompassing medications, dietary control, nutritional supplements, and CAM, can elicit distinct mechanisms to ameliorate renal anemia and enhance kidney function. These mechanisms include heightened EPO production, increased gastrointestinal absorption of iron, elevated iron concentrations, reduction in FGF-23 and hepcidin levels, inflammation inhibition, and stabilization of HIF-2α. The existing evidence underscores the potential applications of these therapeutic approaches. Furthermore, a growing body of research suggests a correlation between the intestine and renal anemia, emphasizing the role of dietary regulation and modulation of intestinal microbiota in mitigating the severity of renal anemia. Recent studies indicate that improving renal anemia levels in CKD can positively impact patient survival by mitigating mortality and pathological consequences associated with CKD. While certain Chinese medicines exhibit nephroprotective and renal anemia-improving properties, caution is warranted as some Chinese medicines may have detrimental effects on the kidneys. Notably, an increasing number of studies propose that the synergistic effects of combining Western medicine with Chinese medicine may more effectively address renal anemia and reduce reliance on Western medications like EPO. Additionally, dietary control, nutritional supplements, and CAM have fewer side effects than traditional treatments, making it a safer option for prolonged use. However, additional large-scale trials are imperative to validate their efficacy in improving renal anemia and elucidating the associated mechanisms.
